# Potential Uses of Biomonitoring Data: A Case Study Using the Organophosphorus Pesticides Chlorpyrifos and Malathion

**DOI:** 10.1289/ehp.9062

**Published:** 2006-06-12

**Authors:** Dana B. Barr, Jürgen Angerer

**Affiliations:** 1 National Center for Environmental Health, Centers for Disease Control and Prevention, Atlanta, Georgia, USA; 2 Institute of Occupational, Social, and Environmental Medicine, Erlangen University, Erlangen, Germany

**Keywords:** biomonitoring, blood, chlorpyrifos, exposure, exposure assessment, human, malathion, risk assessment, urine

## Abstract

**Background:**

Organophosphorus pesticides such as chlorpyrifos and malathion are widely used insecticides. They do not bioaccumulate appreciably in humans and are rapidly metabolized and excreted in the urine. In nonoccupational settings, exposures to these pesticides are typically sporadic and short-lived because the pesticides tend to degrade in the environment over time; however, dietary exposures may be more chronic. Biologic monitoring has been widely used to assess exposures, susceptibility, and effects of chlorpyrifos and malathion; thus, the information base on these compounds is data rich. For biomonitoring of exposure, chlorpyrifos and malathion have been measured in blood, but most typically their urinary metabolites have been measured. For assessing early effects and susceptibility, cholinesterase and microsomal esterase activities, respectively, have been measured.

**Objectives:**

Although many biologic monitoring data have been generated and published on these chemicals, their interpretation is not straightforward. For example, exposure to environmental degradates of chlorpyrifos and malathion may potentially increase f urinary metabolite levels, thus leading to overestimation of exposure. Also, the temporal nature of the exposures makes the evaluation of both exposure and effects difficult. We present an overview of the current biomonitoring and other relevant data available on exposure to chlorpyrifos and malathion and the use of these data in various environmental public health applications.

Organophosphorus (OP) pesticides are phosphate esters comprising a central phosphate atom and three organic side chains, two of which are usually ethyl or methyl and one of which is more specific for a given pesticide. Most OP pesticides registered for use in the United States are used as insecticides. In 1999, 60 million pounds of OP pesticides were used in agriculture and about 17 million pounds were used in nonagricultural applications [U.S. Environmental Protection Agency (U.S. EPA) 2003a, 2003b].

Chlorpyrifos (Figure 1), the most widely used OP insecticide (U.S. EPA 2003a, 2003b), is used to control cutworms, root-worms, termites, and other pests (Kamrin 1997). It is registered for use on a variety of food crops, including grain, cotton, field, fruit, nut, and vegetable crops. Until late 2000, chlorpyrifos was also a commonly used residential pesticide for fire ants, cockroaches, and other household pests. In December 2000 all residential uses of chlorpyrifos, except for preconstruction termite applications, were canceled, and its production for these uses was stopped (U.S. EPA 2002). In February 2001 residential formulation production was stopped, and in December of the same year, all retail sales for residential applications were terminated.

Malathion (Figure 1) is one of the few OP insecticides that still retains residential-use registrations (U.S. EPA 2003a). Malathion is used to control sucking and chewing insects on fruits and vegetables, mosquitoes, other household pests, and animal parasites (Kamrin 1997). It has been used extensively in public health applications in the United States to control the spread of mosquito-borne diseases such as West Nile disease.

We present an overview of the current biomonitoring and other relevant data available on exposure to chlorpyrifos and malathion and the use of these data in various environmental public health applications. Chlorpyrifos and malathion were chosen as case-study examples because they have short residence times in the body and are converted to multiple metabolites *in vivo* that are excreted in the urine. In addition, chlorpyrifos and malathion provide clear examples of the complexities involved with interpreting biomonitoring data, even though the literature base of these chemicals is relatively rich.

## Pharmacokinetics

**Figure 2:**
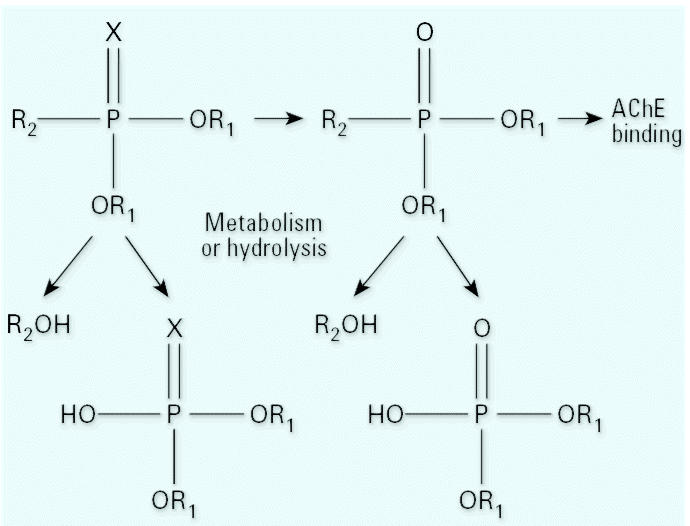
General metabolism and degradation of OP pesticides, where *X* = S or O, R_1_ is typically CH_3_ or CH_2_CH_3_, and R_2_ is an organic moiety unique to the pesticide or pesticide pair. Additionally, side chains (R_2_) with labile functional groups such as esters may hydrolyze to form carboxylic acid metabolites.

Most OP pesticides are believed to undergo a similar metabolism (Barr et al. 2004; Karalliedde et al. 2001; Wessels et al. 2003) (Figure 2). Once they have entered the body, OP insecticides are rapidly metabolized, although a portion may be distributed to and stored in adipose tissue (Kamrin 1997). OP pesticides can be enzymatically converted to their oxon form, which then reacts with available cholinesterase. The oxon also can be enzymatically or spontaneously hydrolyzed to form a dialkyl phosphate (DAP) metabolite and an organic metabolite with the structure of the leaving group. For example, chlorpyrifos can be metabolized to form diethylphosphate (DEP) and 3,5,6-trichloro-2-pyridinol (TCPY). If the pesticide is not converted to its oxon form, it can undergo a hydrolysis to its organic group metabolite and dialkylthionate metabolites. For chlorpyrifos, these metabolites are diethylthiophosphate (DETP) and TCPY (Nolan et al. 1984). These metabolites or their glucuronide or sulfate conjugates are excreted in urine.

Some OP pesticides are metabolized differently than by the methods noted above. Although malathion can undergo metabolism similar to chlorpyrifos to form dimethylphosphate (DMP), dimethylthiophosphate (DMTP), or dimethyldithiophosphate (DMDTP), it likely first undergoes a simple or enzymatic hydrolysis of one or both of the ethyl ester moieties on its alkyl side chain (Bouchard et al. 2003; Krieger and Dinoff 2000). This preliminary hydrolysis of the side chain renders metabolites [i.e., malathion monocarboxylic acid (MMA) and malathion dicarboxylic acid (MDA)] that are appreciably excreted in urine.

In a human pharmacokinetic study, the chlorpyrifos doses administered either orally or dermally resulted in the excretion of TCPY in the urine of participants (Nolan et al. 1984). About 70% of the oral dose of chlorpyrifos was recovered in the urine. Only about 3% of the dermal dose was recovered in the urine, although likely because of poorer absorption by the body via this route. Another report states that about 93% of the chlorpyrifos oral dose is recovered as urinary DAP metabolites, whereas about 1% of the dermal dose was found in urine (Griffin et al. 1999). In an oral dosing study of rats, chlorpyrifos did not accumulate in tissues other than fat (Bakke and Price 1976).

The half-lives of chlorpyrifos in blood (Nolan et al. 1984), adipose tissue (Kamrin 1997), and its metabolites in urine are 24 hr, 62 hr, and 15–30 hr (Griffin et al. 1999; Kamrin 1997; Nolan et al. 1984), respectively (Table 1).

In human volunteers, an average of about 35% of the oral dose of malathion was excreted as the MMA metabolite, whereas MDA represented only about 8% of the total dose and the DAP metabolites represented about 20% of the dose (Bouchard et al. 2003). Another study reported that oral malathion doses were recovered in urine as MMA and MDA in approximately equal proportions (34–39%) and that DMTP was the predominant DAP metabolite (22–65%) (Krieger and Dinoff 2000). In a forensic study evaluating the distribution of malathion among tissues, unmetabolized malathion was detected in the blood and urine of all cases, with the blood level typically higher than that of urine by a factor of 2–3 (Jadhav et al. 1992). Further, malathion was detected in all tissues autopsied, including the liver, spleen, kidney, lung, brain, and muscle, with the highest concentrations in the kidney (Jadhav et al. 1992). The doses in the autopsied cases were much higher than a typical exposure; thus, whether malathion would distribute among these tissues after a low-level exposure is uncertain.

The half-lives of malathion metabolites in urine after dermal, oral, and intravenous administration were 11.8, 3.2, and 4, respectively (Bouchard et al. 2003; Kamrin 1997). Further, the half-life of malathion in blood was estimated to be about 12 min (Bouchard et al. 2003) (Table 1).

## Toxicity Data

The acute toxic effects of OP pesticides result from their ability to inhibit the action of acetylcholinesterase (AChE) in the nervous system, causing a buildup of acetylcholine that results in overstimulation of the nervous system (Karalliedde et al. 2001). These effects are well documented and well understood (Kwong 2002). Because OP pesticides are powerful inhibitors of carboxylic ester hydrolases, including AChE and butyrylcholinesterase, people exposed to high levels of OP pesticides can develop acute cholinergic syndrome, characterized by a variety of symptoms including rhinorrhea, salivation, lacrimation, tachycardia, headache, convulsions, and death (Karalliedde et al. 2001). In addition, these individuals can also develop a proximal and reversible paralysis called intermediate syndrome, organophosphate-induced delayed polyneuropathy, or long-term neurologic sequelae. Symptoms of repeated low-dose exposures in pesticide workers and applicators include impaired memory and concentration, disorientation, severe depression, irritability, confusion, headache, speech difficulties, delayed reaction times, nightmares, sleepwalking, drowsiness, insomnia, and flulike conditions.

Chlorpyrifos is considered moderately toxic and is an EPA class II toxicant [i.e., oral dose that is lethal for 50% of test animals (LD_50_), 50–500 mg/kg] (Kamrin 1997; Tomlin 1997). No teratogenic, mutagenic, or carcinogenic effects have been observed in animal studies; however, a small increase in offspring mortality was observed (Kamrin 1997). Chronic exposure to chlorpyrifos over a 2-year period resulted in increased liver weight in dogs at a dose of 3 mg/kg/day.

Chronic effects of human chlorpyrifos exposures include many of the symptoms of acute toxicity, including plasma and red blood cell cholinesterase depression (Kamrin 1997). In most instances, symptoms subsided after eliminating the exposure. However, newer evidence suggests some persistent neurologic effects after moderate to low-level nonacute exposures (Kamel et al. 2005; Kamel and Hoppin 2004; Young et al. 2005). Exposure to chlorpyrifos *in utero* has been associated with decreased birth weight and length (Perera et al. 2003; Whyatt et al. 2004). Exposure to chlorpyrifos coupled with decreased paraoxonase (PON) activity has been linked to decreased head circumference (Berkowitz et al. 2004).

Malathion is considered slightly toxic and is an EPA class III toxicant (i.e., oral LD_50_ 500–5,000 mg/kg) (Kamrin 1997; Tomlin 1997). No teratogenic or carcinogenic effects have been linked to malathion exposure in test animals; however, rats fed high doses (240 mg/kg/day) of malathion during pregnancy showed an increase in pup mortality; no effect was observed at low doses (Kamrin 1997).

The acute LD_50_ values vary for each pesticide and for each animal model tested. Similarly, the acute and chronic no observed-adverse effect levels (NOAELs) for plasma cholinesterase depression vary for each pesticide. Summarized toxicity information is shown in Table 1.

## Biomarker Characterization

An evaluation for biomarkers of chlorpyrifos and malathion is given in Tables 2 and 3. Aside from PON activity and AChE activity, the biomarkers of chlorpyrifos and malathion are different from each other but, for the most part, are not unique from other OP insecticides. The di- and monocarboxylic acid metabolites of malathion are unique to malathion exposures.

### Markers of exposure

Urine is the most common matrix used for biologic monitoring of OP insecticide exposure, primarily because of its ease of collection and general abundance (Barr et al. 1999). Also, the methodology for urinary metabolite measurements is further developed than for other matrices and thus is more widely available.

Of the OP pesticides registered with the U.S. EPA for use in the United States, about 75% metabolize to form from one to three of the six DAP metabolites (Barr et al. 2004): DMP, DMTP, DMDTP, DEP, DETP, and diethyldithiophosphate. Pesticide-specific information cannot be derived from the quantitative measurement of these metabolites; however, a cumulative dose measure of OP insecticides as a class of pesticides may be obtained. When coupled with information on the known use of certain pesticides such as malathion or chlorpyrifos, the parent OP insecticide to which one was exposed may be inferred. However, total toxicity cannot be derived from these measurements because each metabolite has multiple pesticide sources, each with different toxicities.

The pesticide-specific metabolites are also measured in urine (Barr and Needham 2002; Koch and Angerer 2001). The quantitative measurement of these metabolites provides a measure of dose for a specific pesticide. For example, the measurement of TCPY provides dose information specific to chlorpyrifos or chlorpyrifos methyl. Because the metabolites represent each half of the chlorpyrifos molecule, TCPY and the DAP metabolites of chlorpyrifos are produced in approximately an equimolar ratio. The TCPY and DAP measurements should not be summed to assess chlorpyrifos exposure because the exposure would be overestimated by a factor of 2. However, malathion is unique in that it forms metabolites that do not represent both halves of the molecule. Thus, malathion’s metabolites can be summed as their molar equivalents to evaluate total malathion exposure, if the presence of DAPs in the urine is reasonably certain to be attributable to malathion exposure (e.g., for malathion applicators) (Krieger and Dinoff 2000).

Urinary measurements have several limitations. Temporal variability of measurements in spot urine samples has been documented (MacIntosh et al. 1999) in some studies but has been shown to be more stable in other studies (Meeker et al. 2005). In addition some researchers report more stable or representative measurements are obtained from first morning void collections rather than spot samples collected at other times of the day (Kissel et al. 2005). Thus, in smaller studies or in longitudinal studies, the temporal variability of measurements should be considered. In larger cross-sectional studies, however, a large number of samples may minimize the overall variability of the population providing more representative data.

Monitoring OP pesticide concentrations in blood or blood products offers several advantages (Barr et al. 1999). The parent compounds, instead of their metabolites, which are usually measured in urine, can be directly monitored in blood. This information is especially beneficial because not all OP pesticides are equally toxic. Blood measurements provide an estimation of the dose available for the target site, allowing for prediction of dose–response relationships.

The major disadvantages related to blood measurements are the venipuncture and associated risks (e.g., bruising, discomfort) required to obtain the sample and the analytical challenge of measuring low toxicant concentrations. If available, umbilical cord blood can overcome some of these concerns for measuring recent *in utero* exposures because venipuncture is not needed and relatively large quantities of blood (> 30 mL) can be collected. The invasive nature of venipuncture puts some limits on researchers’ ability to obtain samples from children and pregnant women or to get high participation rates in large-scale studies. In addition, the amount of blood available to perform the analysis is often limited; therefore, ultrasensitive analytical techniques may be required. Analysis is further complicated by the inherently low concentrations of OP pesticides present in the blood (typically seen in the nanogram per liter or parts per trillion range) compared with urinary metabolite concentrations (typically seen in the microgram per liter or parts per billion range) (Barr et al. 1999, 2002).

### Markers of effect

Cholinesterase monitoring has the advantage of providing a measure of physiologic response, but it is a less sensitive marker of exposure (He 1999). In addition, internal interperson variation and variation due to exogenous factors such as pregnancy, disease, co-exposures, and illegal drug use, make interpretation of cholinesterase depression results difficult (Bissbort et al. 2001; Lessenger and Reese 1999).

AChE measurements have been used extensively in occupational monitoring of pesticide applicators (Magnotti et al. 1988) but have not been widely used in general population exposure studies, primarily because of their insensitivity to lower level exposures.

## Methodology

### Urine analytic methods

Many methods have been reported in the literature for the measurement of TCPY, MMA, MDA, and the nonspecific DAP metabolites. Methods for measuring the six nonspecific DAP metabolites are the most common (Aprea et al. 1996; Barr and Needham 2002; Bradway and Shafik 1977; Bravo et al. 2002, 2004; Hardt and Angerer 2000; Hernandez et al. 2002; Moate et al. 1999; Shafik et al. 1973). These methods use liquid–liquid extraction with polar solvents such as ethyl acetate or diethyl ether, cyclohexyl solid-phase extraction, azeotropic distillation, or lyophilization to isolate the DAPs from the urine matrix. Methods using a variety of reagents, most often pentafluorobenzyl bromide (PFBBr), derivatize the DAPs. Those methods that derivatize using methylating agents such as diazomethane cannot obtain an accurate analysis of DMP because endogenous inorganic phosphate produces the same trimethyl derivative. The derivatized extracts are analyzed using gas chromatography (GC) coupled with flame photometric detection (Aprea et al. 1996; Moate et al. 1999), flame ionization detection, mass spectrometry (MS) (Hardt and Angerer 2000), or tandem mass spectrometry (MS/MS) (Bravo et al. 2002, 2004; Hernandez et al. 2002). Many of these methods have limits of detection (LODs) in the mid-microgram per liter (ppb) range, but several can detect levels in the low microgram per liter range (Aprea et al. 1996; Hardt and Angerer 2000; Moate et al. 1999) or sub-microgram per liter range (Bravo et al. 2002, 2004). Additionally, a high-performance liquid chromatography (HPLC)–MS/MS-based method using online solid-phase extraction has been reported, although the LODs are higher than achievable using the more traditional methods (Hernandez et al. 2002).

Methods that measure TCPY usually include an acid or enzyme hydrolysis followed by solid-phase or a liquid–liquid extraction (Barr et al. 1999). The extracted analytes are then derivatized, with the most popular derivatizing agents being PFBBr and diazomethane. The derivatized analytes are analyzed using GC–electron capture detection, GC-MS (Koch and Angerer 2001), and GC-MS/MS (Hill et al. 1995). Alternatively, the underivatized TCPY can be analyzed using HPLC, HPLC–electrospray ionization–MS/MS (Olsson et al. 2003), or HPLC–atmospheric pressure chemical ionization–MS/MS (Olsson et al. 2004). The LODs of these methods vary, but many are suitable for measuring TCPY resulting from incidental exposures.

MMA and MDA are either measured as the intact metabolite using HPLC-MS/MS (Baker et al. 2000; Beeson et al. 1999; Olsson et al. 2004) or subjected to a base hydrolysis to form DMP and DMTP. The DMP and DMTP can then be analyzed using the DAP methodology (Bradway and Shafik 1977; Fenske and Leffingwell 1989). The LODs of these methods vary, but many are suitable for measuring metabolites resulting from incidental exposures.

### Blood analytic methods

#### Biomarkers of exposure

Several laboratory methods have been reported that measure intact OP pesticides in blood (Fournier et al. 1978; Frenzel et al. 2000; Kawasaki et al. 1992; Liu et al. 1989). These methods employ a range of analytical techniques that directly affect both the sensitivity and selectivity of the analysis. Generally, MS-based techniques are able to measure lower levels of the insecticides and are more selective in their measurements (e.g., reduce false positives, eliminate interfering components). The vast majority of these methods were developed for forensic applications or for diagnosis of acute pesticide intoxication and have LODs in the microgram per liter to milligram per liter range—levels unsuitable for detection of incidental exposures. For example, Frenzel et al. (2000) reported a method to measure methamidaphos and methyl parathion in blood with LODs of about 25 μg/L. However, data reported by Whyatt et al. (2003) from their northern Manhattan/ Harlem minority birth cohort indicate that OP insecticide levels in pregnant women and cord blood were about three orders of magnitude lower. Recent advances in analytical instrumentation have facilitated the development of highly sensitive methods (Barr et al. 2002); however, these methods are often complicated and costly, potentially precluding their use for routine analysis.

#### Biomarkers of effect

The electrometric and colorimetric methods, which measure change in pH and light absorbance, respectively, most often measure AChE suppression. Both methods measure serum and erythrocyte cholinesterases and are relatively simple, inexpensive, and reproducible (Vandekar 1980). Even with modern testing kits and methods, the determination of serum and erythrocyte AChE activity levels is highly dependent on technician experience and skill.

### Uncertainties in measurements

Aside from the obvious limitations involved in the measurement process (e.g., laboratory imprecision, contamination), other forms of uncertainties in the biomonitoring data should be considered.

Selectivity can refer to the ability of a measurement technique to differentiate a single analyte that is measured from other components of the matrix (i.e., reducing false positives). Or, selectivity can refer to the ability of the analyte measured to accurately and unequivocally identify exposure to the target chemical of interest. The former can be easily achieved by the judicious use of chromatography to resolve the analytes in time and by the use of a selective detection technique such as MS, electron capture detection, or nitrogen phosphorus detection. Generally, MS-based methods are regarded as the most selective measurement techniques currently available; however, these techniques are often complex and costly and require specialized training for operation (Barr and Needham 2002). Furthermore, the cost of the instrumentation often precludes their use by many laboratories, thus hindering methodology transfer and laboratory capacity building. Nonetheless, the data generated using these methods are typically less prone to false-positive analyses (Barr et al. 1999). Alternative methods such as immunoassays (MacKenzie et al. 2000; Shackelford et al. 1999) and less specialized technologies may be employed to reduce costs or increase analytical throughput. If alternative methods are used, a harmonization of the various methods should be performed, as discussed above, to ensure that data generated using different methods are comparable.

The selectivity of the analyte measured to accurately reflect the exposure of interest does not depend on the measurement technique but rather on the biomarker that is measured. Chlorpyrifos can best illustrate this point. Chlorpyrifos can be metabolized to DEP and DETP, which are also common to many *O*,*O*-diethyl–substituted OP pesticides such as diazinon. Further complicating the issue, the DAPs may be present in environmental media as the environmental degradates of the pesticides. Thus, if DEP and DETP are detected in the urine sample, one could conclude only that exposure to an *O*,*O*-diethyl–substituted pesticide or its environmental degradate has occurred. However, additional data such as nearby pesticide application, or prevalence of pesticide use, could be used to help deduce that an exposure to chlorpyrifos has occurred. Nonetheless, the measurements indicate unequivocally only that an exposure to the *O*,*O*-diethyl OP pesticides or their degradates has occurred.

The organic metabolites of chlorpyrifos are TCPY and its conjugates. These metabolites are also common to chlorpyrifos methyl. Additionally, TCPY can be derived from exposure to its environmental degradates: the oxons of either chlorpyrifos or chlorpyrifos methyl and TCPY itself. Thus, urinary TCPY can indicate exposure to several different chemicals, including environmental TCPY; therefore, it may not be an appropriate bio-marker for low-level exposures to chlorpyrifos, especially when TCPY dominates the environmental media (Morgan et al. 2004).

The only way to unequivocally identify chlorpyrifos exposure is by measuring the intact pesticide in blood samples because the intact pesticide is not appreciable in urine. However, blood measurements are inherently difficult because the levels are typically about three orders of magnitude lower than urinary metabolite measurements; thus, highly sensitive analytical techniques must be used, which in turn generally drive up the cost of analysis. These techniques are not available for many of the OP insecticides; thus, total class exposure information would be difficult to obtain. Furthermore, OP pesticides are less stable in blood than their metabolites are in urine; therefore, special precaution to prevent degradation must be used.

### Temporal variability in spot urine samples

The variability of OP insecticide metabolite concentrations in samples collected from an individual over time is of concern. If a single sample taken at a given point in time cannot accurately assess a person’s average exposure over a given time frame, then multiple samplings are necessary, although this can become costly and burdensome to the participant. Temporal variability can include the variation of a given chemical in multiple samples collected on a single day or can include variation among days, months, or seasons. How accurately can a single sample represent a day’s exposure to a given chemical, or how accurately can a single sample represent a person’s exposure over a longer period of time? These questions can be more easily answered for chronic exposures because the exposure is repeated; thus, the amount in a given sample would likely be representative of that average exposure. However, for episodic exposures, which is likely the case with OP insecticide exposures, the questions become more difficult to answer and may vary from pesticide to pesticide. For urine matrix a 24-hr urine sample is preferred, rather than a single spot sample on a given day; however, this is very burdensome for the participant and is often logistically difficult. If a 24-hr sample cannot be obtained, a first-morning void is often preferred because the urine is more concentrated and the collection represents a longer window of accumulation (usually > 8 hr); thus, the analyte level is more likely to reflect an average daily concentration. Regardless of whether the sample collected is a first morning void, if the sample does not represent a full 24-hr collection, the variability in urine concentrations (i.e., degree of dilution of the urine components as a result of water intake) must be considered.

To evaluate daily, monthly, or seasonal variations of analyte in urine, sequential samples are often taken days or weeks apart to evaluate the intraindividual variation over time and to determine whether an accurate classification of exposure is possible from a single spot sample. Several studies have evaluated the weekly or seasonal variation for certain pesticide metabolites in urine, although most pesticide metabolites have not been studied. The existing data indicate that a single sample is usually not sufficient to accurately quantify exposure to the target pesticide; thus, multiple samples must be collected over time (MacIntosh et al. 1999). Newer data, however, suggest a single sample may allow a broad classification of exposure (Meeker et al. 2005). Further studies to indicate the variability of commonly measured analytes in urine should be conducted to ensure that multiple samples are required for the target analyte(s), thus reducing cost, and to better establish the most appropriate sampling time frame (e.g., collect samples every fourth day). In all likelihood, sampling for nonpersistent chemicals will require multiple samples taken over the course of the study at regular intervals (e.g., weekly, monthly, semiannually).

## Exposure Assessment

Exposure assessments for OP pesticides have used historical or observational measurements, multimedia environmental measurements, and biomonitoring measurements. In addition, models have been developed to predict OP pesticide exposures. The models rely primarily on an understanding of product use, measurements of OP pesticides in various media, estimates of human contact, and pharmacokinetic assumptions based on animal and human data. The model estimates have typically been validated using experimental data from bio-monitoring. Experimental data using questionnaires or environmental, personal, and biologic monitoring have provided a wealth of exposure assessment data, although with some limitations.

### Historical or observational measurements

Four types of historical or observational instruments are used to collect data on chlorpyrifos or malathion exposure: *a*) questionnaires including product use information, *b*) time and activity logs, *c*) diaries of specific activities such as foods eaten, and *d*) visual assessments, database inventories, and check lists. Although data collected from these instruments have not always been reliably linked with biomonitoring data, they have been used frequently, in conjunction with biomonitoring data, to evaluate predictors of exposure.

### Multimedia environmental measurements

Biomonitoring measurements are limited in that they provide little if any information on the route or pathway of exposure; however, in many instances, this is seen as an advantage because all routes or pathways are integrated so that only one exposure measure is needed. Further, typical biomonitoring measurements from single spot samples provide no information on the frequency, magnitude, and duration of exposures. Thus, many studies have evaluated chlorpyrifos or malathion exposure using measurements in multiple environmental and personal matrices such as air, water, duplicate diet, and dust. A single measurement in these matrices, as with biomonitoring measurements, provides a snapshot estimate of the exposure as the chlorpyrifos or malathion levels may change in the matrices over time. The measurements, when coupled with historical and observational data, may allow the calculation of the frequency, magnitude, and duration of exposures. In addition, these measurements can be used as an input into deterministic models to estimate total exposure. Increasingly, environmental measurements used in exposure assessment are also augmented with biomonitoring measurements.

### Biologic measurements

#### Blood measurements

Few studies have focused on biomonitoring measurements of chlorpyrifos or malathion in blood primarily because of the limitations of blood as a matrix as outlined above. Maternal and cord blood plasma have been used to evaluate fetal exposures to chlorpyrifos. The plasma concentrations of chlorpyrifos ranged from < 1 to 10 pg/mL (Whyatt et al. 2003, 2004). Cord and maternal plasma chlorpyrifos levels were highly correlated (Whyatt et al. 2003). Pooled serum samples had a mean chlorpyrifos concentration of 9 pg/mL (Barr et al. 2002). Chlorpyrifos has been detected at somewhat higher levels in citrus farmers. In addition, malathion has been detected in postmortem blood samples from a fatal poisoning case at very high levels (1.9–517 μg/mL) (Jadhav et al. 1992).

A potentially novel marker of OP insecticide exposure in blood is the measurement of biomolecular adducts of OP insecticides. Fidder et al. (2002) reported a technique for retrospectively detecting exposure to OP nerve agents by measuring a specific nonapeptide that represents the adducted portion of butryl-cholinesterase. They demonstrated its potential applicability to OP insecticides; thus, similar methods are being developed for OP insecticides. These measurements offer the advantage of a longer term dosimeter of exposure using potentially an early marker of effect.

#### Urinary metabolite measurements

Most biologic monitoring measurements assessing chlorpyrifos or malathion exposure have involved the measurement of their urinary metabolites. The urinary metabolite levels of various occupational and nonoccupational studies have been previously reviewed (Barr et al. 2004, 2005). Concentrations of the urinary metabolites reported in the literature vary greatly depending on the exposure scenario but usually hover in the low nanograms per milliliter range for typical background exposures (Barr et al. 2004, 2005). Because urinary TCPY can also be derived from exposure to environmental TCPY or other sources, it may not be a good marker of chlorpyrifos exposure (Morgan et al. 2004). Urinary MMA and MDA have been less frequently measured as the direct metabolites. Of these, MDA has been measured the most often because of its greater analytical stability compared to MMA.

## Environmental Public Health Uses of Chlorpyrifos and Malathion Biomonitoring Data

### Identification of prevalent exposures

To evaluate the prevalence of exposure to either chlorpyrifos or malathion, existing biomonitoring data must be coupled with information about the relative contribution of environmental degradates to the overall urinary metabolite levels. To properly evaluate the prevalence of exposures, we must have a better understanding of the environmental degradate contribution from the various exposure matrices and that pathway(s) of exposure predominates. Further, a more complete picture of OP insecticide exposure could be obtained if pesticide-specific biomonitoring data were available for more OP insecticides. Regardless, the existing data can provide a reasonable upper-bound estimate of the prevalence of exposures to chlorpyrifos and malathion.

### Evaluation of trends in exposure

Assuming that environmental degradate contributions to urinary levels remain relatively constant over time that may or may not be the case, urinary measurements taken over time should prove suitable for evaluating temporal trends in exposure. Biomarker levels would represent a maximum exposure level. Significant decreases in these levels over time would suggest that exposures to the parent chemical and/or degradates or even the use of these pesticides were reduced. For chlorpyrifos evaluation, one should recognize that a percentage of the TCPY may be also from exposure to chlorpyrifos methyl; thus, if the relative use of these two pesticides remains constant, temporal trend evaluations should be valid. However, if the use patterns are moving in opposite directions (i.e., chlorpyrifos use is decreasing, but chlorpyrifos methyl use is increasing), true trends in exposure to one of the chemicals may be masked. A particular advantage of the large population-based studies such as the National Health and Nutrition Examination Survey (NHANES) is that the sample size is sufficient and the participant selection is completely random such that the “noise” in the data set due to unusual exposures not typical of the general population should be minimized. Blood chlorpyrifos measurements alone should be suitable for evaluation of temporal trends in chlorpyrifos exposures if background levels are measurable with existing methodology.

### Identification of unusually exposed population subgroups

The existing biomonitoring data are sufficient to evaluate unusually exposed population subgroups as long as the subgroups have been defined at the study level and the relative contribution to the measurements from exposure to the environmental degradates is known. For example, the NHANES database is sufficient to identify the most highly exposed demographic groups defined in the study; however, certain demographic information such as geographic subgroups or infants are not a part of the study or are not a defined variable for analysis. To augment these data, targeted studies looking at similar exposures in these populations of interest should be conducted. Alternatively, the NHANES study could be expanded to include these additional population groups, although this may not be logistically feasible.

### Provide reference range data for comparison

The existing population data provide adequate information from which reference range concentrations can be derived. To supplement these data, exposure assessment or health effects studies can also enroll control subjects to provide reference data for their specific geographic region.

### Evaluation of effectiveness of an intervention or regulatory action

Assuming that environmental degradate contributions to urinary levels remain relatively constant over time (e.g., 80% of TCPY from dietary exposure is from environmental TCPY), urinary measurements taken over time should prove suitable for evaluating the effectiveness of regulations, providing “background” or pre-regulatory levels were determined. Blood measurements should be suitable on their own to evaluate the effectiveness of regulatory actions on reducing exposures.

### Risk assessment

Urinary biomonitoring measurements have been used in risk assessment approaches for both chlorpyrifos and malathion; however, these data alone are not sufficient for risk assessment. Data on the magnitude, duration, and frequency of exposures should be obtained, likely through environmental measurements. In addition, detailed human pharmacokinetic information is required to appropriately evaluate the various biologic compartments into which the chemicals will be deposited or eliminated. Animal and *in vitro* toxicity (or other health end point) information should be used in conjunction with the human exposure data to perform the risk assessment.

## Conclusions

Biomonitoring of exposure to OP insecticides, primarily for chlorpyrifos and malathion, has been performed extensively over the last 30 years, providing a wealth of data for evaluation. However, these data should be used cautiously for each intended application, and the uncertainties associated with the measures should be acknowledged. Larger gaps in data required to use biomonitoring data for a given purpose may produce more uncertainties in the interpretation; however, biomonitoring data alone can serve a better purpose than making public health decisions without any human data. Although much data exist, we can recommend several research activities that would enhance the utility of the existing database and reduce the uncertainties associated with its use:

- Perform additional multimedia exposure assessments to better define relative contribution of environmental degradates to urinary metabolite concentrations (e.g., preformed DAPs or TCPY in the environment). Include additional OP insecticides in the assessments to address combined toxicity issues.
- Develop methods to measure all urinary pesticide-specific metabolites of OP insecticides used in the United States to allow differentiation of effects specifically due to chlorpyrifos or malathion exposures.
- Develop methods to provide blood pesticide measurements for all OP insecticides used in the United States to allow differentiation of effects specifically due to chlorpyrifos or malathion exposures.
- Develop methods to evaluate biomolecular adducts of OP insecticides to provide a longer term dosimeter of exposure.
- Perform exposure assessment studies looking at targeted populations of interest such as children, general population of defined geographic regions, and pregnant women.
- Perform chronic health effects studies in animals at doses similar to those encountered in human exposure scenarios.

## Figures and Tables

**Figure 1:**
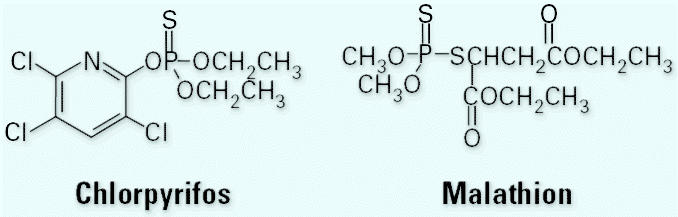
The structures of chlorpyrifos and malathion.

**Table 1:** What information do we currently have for biomonitoring of exposure to a given OP pesticide?

Data	Chlorpyrifos	Malathion
Biomarkers of exposure	TCPY, DEP, DETP, chlorpyrifos oxon	MMA, MDA, DMP, DMTP, DMDTP, malathion
Biomarkers of effect	AChE depression	AChE depression
Biomarkers of susceptibility	PON genotype/phenotype	PON genotype/phenotype
Matrices used	Urine, blood (serum, plasma, whole)	Urine, blood (serum, plasma, whole)
Pharmacokinetics (human)	Well defined	Well defined
Interindividual variation in pharmacokinetics	Not well understood	Not well understood
Temporality of marker	Short-lived: blood half-life, 6 hr; urine half-life, 15–24 hr	Short-lived: blood half-life, 12 min; urine half-life, 3–12 hr
Temporality of exposure	Potential chronic low level in diet; episodic low-level acute exposures	Potential chronic low level in diet; episodic low-level acute exposures
Specificity of biomarker	See Table 2	See Table 3
Stability of the marker	See Table 2	See Table 3
Primary route of environmental exposure	Diet	Diet
Mass balance (has anyone attempted to balance deterministic measures with biomarker measures?)	For TCPY environmental exposures, mass balance has not been achieved	For MMA and MDA occupational exposures, mass balance achieved
Biologically active agent	Chlorpyrifos oxon	Malathion oxon
Known animal toxicity (acute)	Oral LD_50_, 8–2,000 mg/kg; percutaneous LD_50_, 2,000 mg/kg or greater; inhalation LD_50_, > 0.2 mg/L for 4–6 hr	Oral LD_50_, 167–3,320 μg/kg/day; percutaneous LD_50_, 4,100 mg/kg; increased pup mortality at 200 mg/kg/day
Known animal toxicity (chronic)	3 mg/kg/day for 2 years for increased liver weight	Not known
Known human toxicity (acute)	Oral NOAEL, 100 μg/kg/day	Oral NOAEL, 201 μg/kg/day
Known human toxicity (chronic)	Oral NOAEL, 30 μg/kg/day	Not known
Known mechanism of toxicity	AChE inhibitors	AChE inhibitors
Human health associations	Acute effects (e.g., headache, dizziness, difficult breathing, excessive salivation, low birth weight, low birth length, death, OIDN)	Acute effects (e.g., headache, dizziness, difficult breathing, excessive salivation, convulsions, death, OIDN)
Agreement among studies	Agreement with acute effects; conflicting outcomes with epidemiology studies that used different biomarker measures	NA
Mixtures/synergism	Synergism of effects on birth outcome when diazinon combined with chlorpyrifos	NA
Link between human/animal toxicity and dose?	Yes	Yes
Link between human/animal dose and biomarker?	No	No

Abbreviations: NA, not applicable; OIDN, organophosphate-induced delayed neuropathy.

**Table 2:** Evaluation of biomarkers for chlorpyrifos.

Validation parameter	CP	CPO	TCPY	DEP	DETP	AChE	PON
Specificity of marker for exposure^a^	1	1	2	3	3	3	NA
Matrix for measurement	Bl	Bl	U	U	U	Bl	Bl
Alternative exposures that may result in presence of biomarker in matrix	None	A	A, B, chlorpyrifos methyl	A, B, D	A, B, D	A, C, D, E, nerve agents	NA
Specificity of marker for predicting health outcome^a^	2	2	3	3	3	1	2
Stability of marker^b^	2	2	1	1	1	1	1
Data from multiple labs	No	No	Yes	Yes	Yes	Yes	Yes
Interlaboratory comparisons	No	No	Yes	Yes	Yes	For some	No

Abbreviations: A, environmental oxon; B, environmental degradates; Bl, blood; C, *O*,*O*-dimethyl–substituted OP pesticides; CP, chlorpyrifos; CPO, chlorpyrifos oxon; D, *O*,*O*-diethyl–substituted pesticides; E, other OP and carbamate pesticides; NA, not applicable; U, urine;

a 1, Most specific; 2, relatively specific with some limitations; 3, nonspecific.

b 1, Very stable; 2, unstable.

**Table 3:** Evaluation of malathion biomarkers.

Validation parameter	MTN	MTNO	MMA	MDA	DMP	DMTP	DMDTP	AChE	PON
Specificity of marker for exposure^a^	1	1	2	2	3	3	3	3	NA
Matrix for measurement	Bl	Bl	U	U	U	U	U	Bl	Bl
Alternative exposures that may result in presence of biomarker in matrix	None	A	A, B	A, B	A, B, C	A, B, C	A, B, C	A, C, D, E, nerve agents	NA
Specificity of marker for predicting health outcome^a^	2	2	3	3	3	3	3	1	2
Stability of marker^b^	2	2	1	1	1	1	1	1	1
Data from multiple labs	No	No	Yes	Yes	Yes	Yes	Yes	Yes	Yes
Interlaboratory comparisons	No	No	Yes	Yes	Yes	Yes	Yes	For some	No

Abbreviations: A, environmental oxon; B, environmental degradates; Bl, blood; C, *O*,*O*-dimethyl–substituted OP pesticides; D, *O*,*O*-diethyl–substituted pesticides; E, other OP and carbamate pesticides; MTN, malathion; MTNO, malathion oxon; NA, not applicable; U, urine.

a 1, Most specific; 2, relatively specific with some limitations; 3, nonspecific.

b 1, Very stable; 2, unstable.
